# Meta-Viromic Sequencing Reveals Virome Characteristics of Mosquitoes and *Culicoides* on Zhoushan Island, China

**DOI:** 10.1128/spectrum.02688-22

**Published:** 2023-01-18

**Authors:** Xiaojing Yang, Shiyu Qin, Xiong Liu, Na Zhang, Jiali Chen, Meiling Jin, Fangni Liu, Yong Wang, Jinpeng Guo, Hua Shi, Changjun Wang, Yong Chen

**Affiliations:** a School of Public Health, China Medical University, Shenyang, Liaoning Province, China; b College of Public Health, Zhengzhou University, Zhengzhou, Henan Province, China; c Chinese PLA Center for Disease Control and Prevention, Beijing, China; Connecticut Agricultural Experiment Station

**Keywords:** core virome, *Culicoides*, meta-virome, mosquito, virome characteristics

## Abstract

Mosquitoes and biting *Culicoides* species are arbovirus vectors. Effective virome profile surveillance is essential for the prevention and control of insect-borne diseases. From June to September 2021, we collected eight species of female mosquito and *Culicoides* on Zhoushan Island, China, and used meta-viromic sequencing to analyze their virome compositions and characteristics. The classified virus reads were distributed in 191 genera in 66 families. The virus sequences in mosquitoes with the largest proportions were Iflaviridae (30.03%), Phasmaviridae (23.09%), Xinmoviridae (21.82%), Flaviviridae (13.44%), and Rhabdoviridae (8.40%). Single-strand RNA^+^ viruses formed the largest proportions of viruses in all samples. Blood meals indicated that blood-sucking mosquito hosts were mainly chicken, duck, pig, and human, broadly consistent with the habitats where the mosquitoes were collected. Novel viruses of the *Orthobunyavirus*, *Narnavirus*, and *Iflavirus* genera were found in *Culicoides* by de-novo assembly. The viruses with vertebrate hosts carried by mosquitoes and *Culicoides* also varied widely. The analysis of unclassified viruses and deep-learning analysis of the “dark matter” in the meta-viromic sequencing data revealed the presence of a large number of unknown viruses.

**IMPORTANCE** The monitoring of the viromes of mosquitoes and *Culicoides*, widely distributed arbovirus transmission vectors, is crucial to evaluate the risk of infectious disease transmission. In this study, the compositions of the viromes of mosquitoes and *Culicoides* on Zhoushan Island varied widely and were related mainly to the host species, with different host species having different core viromes. and many unknown sequences in the *Culicoides* viromes remain to be annotated, suggesting the presence of a large number of unknown viruses.

## INTRODUCTION

Mosquitoes and biting *Culicoides* species are common vectors of arboviruses that cause a variety of significant diseases, including dengue virus, Zika virus, Getah virus, West Nile virus, Japanese encephalitis virus, bluetongue virus, and Banna virus (1–12). Apart from arboviruses, many insect-specific viruses (ISVs) carried by mosquitoes and *Culicoides* remain to be discovered and characterized. Some ISVs have shown the potential to modulate arbovirus replication and the vector competence of mosquitoes; for example, the Palm Creek virus, Nhumirim virus, Eilat virus, and Culex flavivirus may inhibit the West Nile, dengue, and chikungunya viruses (13–23).

Viromes carried by mosquitoes have been shown to be species specific (24, 25), and the core virome is related closely to vertical transmission and the environment (26–28). The viruses of the core viromes of various organisms have been characterized and shown to be significantly similar among individuals of the same mosquito species and diverse among species (26, 29). Consistent with vertical virus transmission, the core virome was found to remain stable in laboratory-grown and field-collected Aedes albopictus across developmental stages (26). Comparative analysis of the most abundant viruses has enabled the characterization of the viromes of different species, and the identification of species likely to transmit the same virus (28). *Culicoides* are abundant and can actively or mechanically carry a variety of pathogens (30). Most of the viruses transmitted by *Culicoides* are members of the Reoviridae, Rhabdoviridae, and Perbunyaviridae families (31). However, few studies have been performed to examine *Culicoides* viromes. Meta-viromic approaches have enabled the characterization of viromes carried by *Culicoides* in some regions and promoted the discovery of new viruses. Analysis of the RNA viromes of 10 *Culicoides* species in Thrace, southeastern Europe, led to the identification of novel RNA viruses with genomic signatures and revealed that each *Culicoides* species carried a specific virus set (32). The viromes of *Culicoides impunctatus* populations in Scotland (33), *Culicoides imicola* in Senegal (34), *Culicoides arakawae* in Japan (35), and mixed *Culicoides* populations (6, 36) have been examined.

Next-generation sequencing has greatly facilitated the study of the viromes carried by mosquitoes and *Culicoides*, and accelerated the discovery of new pathogens. The identification of many new RNA and DNA viruses by next-generation sequencing is of great significance for the dynamic monitoring of pathogens and the response to emerging and recurrent infectious diseases (37, 38). In a typical viral group study, however, much of the sequence data, including a significant number of unannotated sequences, is unknown and is referred to as “dark matter.” The advent of computational tools for the identification of viral sequences in metagenomics has improved our ability to identify known and novel viruses. In recent years, deep-learning methods have been used to analyze the presence of dark matter in sequences, which adds understanding of the viral components (33–36) and enables the functionally relevant representation of unknown and unannotated sequences (39). In this study, we attempted to use deep-learning methods to characterize potential viral sequences in mosquitoes and midges.

In the present study, we identified species of mosquitoes and *Culicoides* in five habitats related closely to human activities on Zhoushan Island through molecular biology and morphological analysis. Using meta-viromic sequencing, bioinformatics analysis, and deep learning, we explored the characteristics and compositions of the core viromes of mosquitoes and *Culicoides* on the island. Our findings improve the understanding of the mosquito and *Culicoides* viral spectra and aid the evaluation of the risk of local mosquito- and midge-borne infectious disease occurrence.

## RESULTS

### Samples.

**FIG 1:**
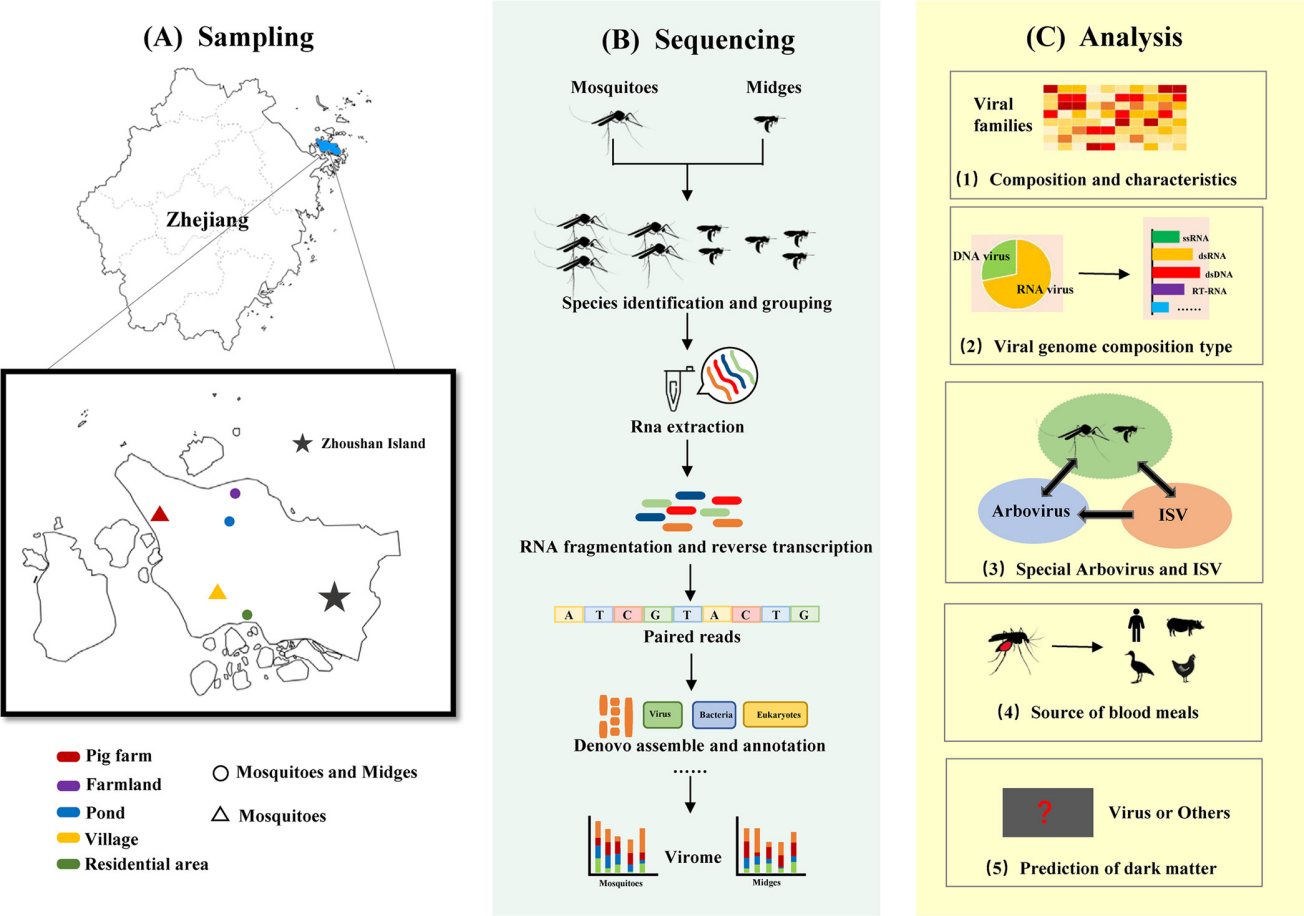
Maps from Ali Cloud data visualization platform. The sampling, sequencing, and comparative viromic analysis of mosquitoes and *Culicoides*. (A) Sampling sites in the main island of Zhoushan city. The map shows the distribution of samples’ location and habitat of five sampling sites in Dinghai district, Zhoushan city, Zhejiang Province, China. (B) Meta-virome sequencing and bioinformatic analysis pipeline of mosquitoes and *Culicoides* samples. (C) Comparative viromic analysis processes of *Culicoides* and mosquito samples.

In total, approximately 12,300 mosquitoes of at least eight species from four genera (*Culex*, *Anopheles*, *Aedes*, and *Armigeres*) were collected at the sampling sites. The dominant mosquito species differed among sites (Table S1). About 5,000 *Culicoides* were collected from the sites; based on their morphological characteristics, three dominant species (*C. arakawae*, *C. lungchiensis*, and *C. punctatus*) were identified (Fig. 1A). Mosquito and midge group and sequence data are provided in Table 1.

**TABLE 1:** Pooling information of mosquitoes and *Culicoides* samples

Pool name	Species	Sample location	Sample count^*a*^	Visible blood engorgement
Midge_1	*Culicoides*	Farmland	30	Unknown
Midge_2	*Culicoides*	Residential area	30	Unknown
Midge_3	*Culicoides*	Pond	20	Unknown
Midge_4	*Culicoides*	Residential area	50	Unknown
Midge_5	*Culicoides*	Residential area	50	Unknown
Ae.a_1	Aedes albopictus	Residential area	8	No
Ae.a_2	Aedes albopictus	Village	4	Yes
Ae.v	*Aedes vexans*	Village	2	No
An.s_1	*Anopheles sinensis*	Pig farm	10	No
An.s_2	*Anopheles sinensis*	Pond	10	No
An.s_3	*Anopheles sinensis*	Pond	10	No
Ar.s_1	*Armigeres subalbatus*	Residential area	1	No
Ar.s_2	*Armigeres subalbatus*	Village	2	Yes
C.p.p_5	Culex pipiens *pallens*	Residential area	1	Yes
C.p.p_6	Culex pipiens *pallens*	Residential area	2	No
C.p_1	Culex pipiens	Farmland	5	No
C.p_2	Culex pipiens	Pig farm	2	No
C.p_3	Culex pipiens	Pond	1	No
C.p_4	Culex pipiens	Residential area	7	No
C.q	Culex quinquefasciatus	Residential area	8	No
C.t_1	*Culex tritaeniorhynchus*	Farmland	10	No
C.t_2	*Culex tritaeniorhynchus*	Village	10	No
C.t_3	*Culex tritaeniorhynchus*	Village	5	No
C.t_4	*Culex tritaeniorhynchus*	Farmland	1	Yes
C.t_5	*Culex tritaeniorhynchus*	Pig farm	10	No
C.t_6	*Culex tritaeniorhynchus*	Pig farm	8	Yes
C.t_7	*Culex tritaeniorhynchus*	Pond	10	No
C.t_8	*Culex tritaeniorhynchus*	Residential area	8	No

a The number of samples in each pool is related to the number of samples collected from each site.

### Virome compositions.

**FIG 2:**
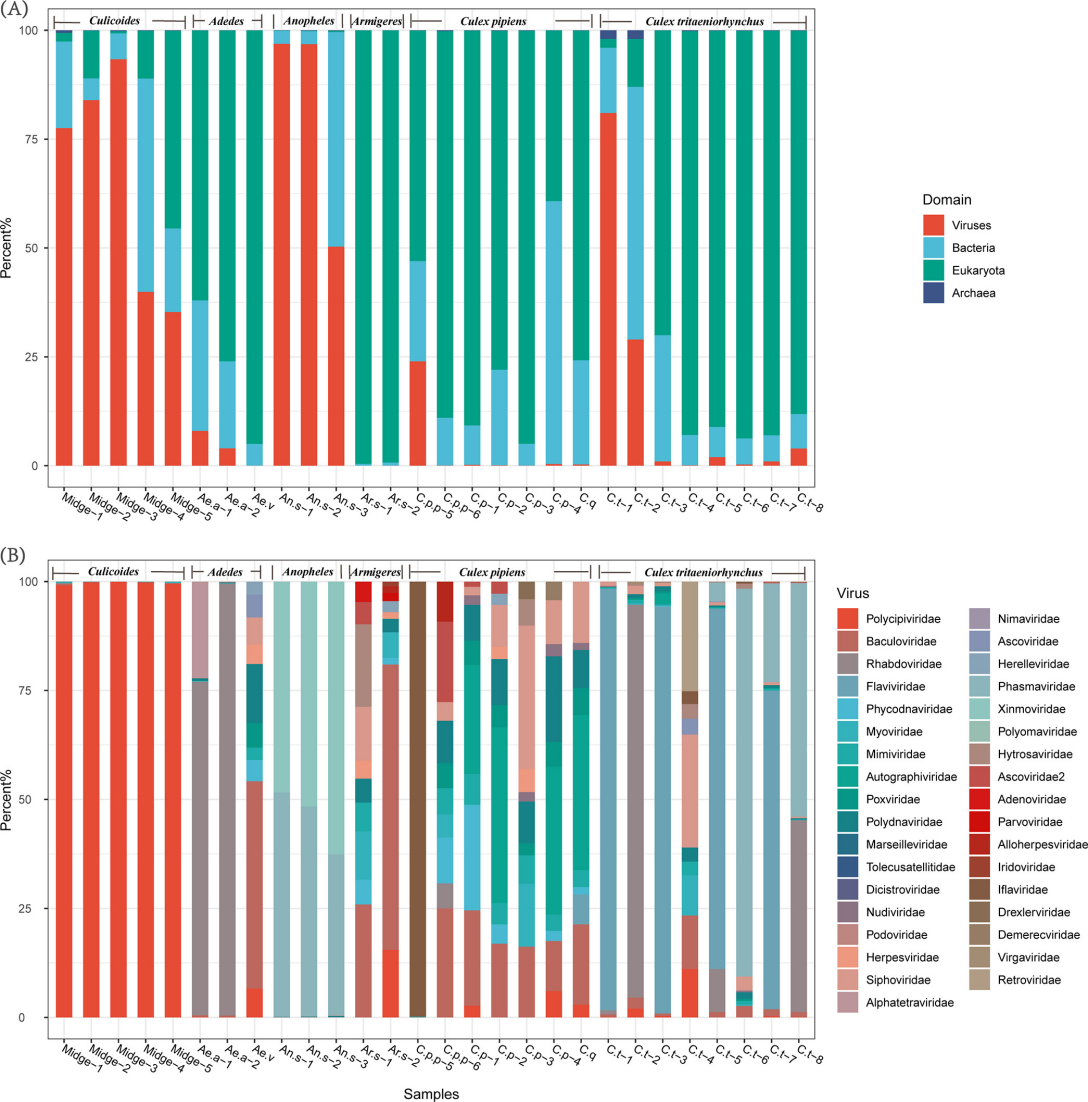
Composition of sequencing reads annotated with Kraken2. (A) The proportion of the sequencing reads annotated to eukaryotes, bacteria, archaea and fungi. (B) The composition of the top 10 classified viral families in each sample pool.

Meta-viromic sequencing of 28 vector pools yielded an average of 9,634,984 (range = 360,702 to 55,161,908) reads per pool for virus discovery and characterization. Reads assigned to viruses represented 0.007% to 97% of all reads in each pool (Fig. 2A). The classified viral reads were distributed in 191 genera of 66 families; 99.67% of them belonged to six families. The largest proportion (93%) belonged to the Polycipiviridae, found mainly in *Culicoides* samples (99.99%). The viral families commonly identified in the mosquito viromes were Iflaviridae (30.03%), Phasmaviridae (23.09%), Xinmoviridae (21.82%), Flaviviridae (13.44%), and Rhabdoviridae (8.40%), found mainly in Culex pipiens *pallens*, *Anopheles sinensis*, *Culex tritaeniorhynchus*, and *Ae. albopictus*, respectively.

### Host species are important drivers of virome composition.

**FIG 3:**
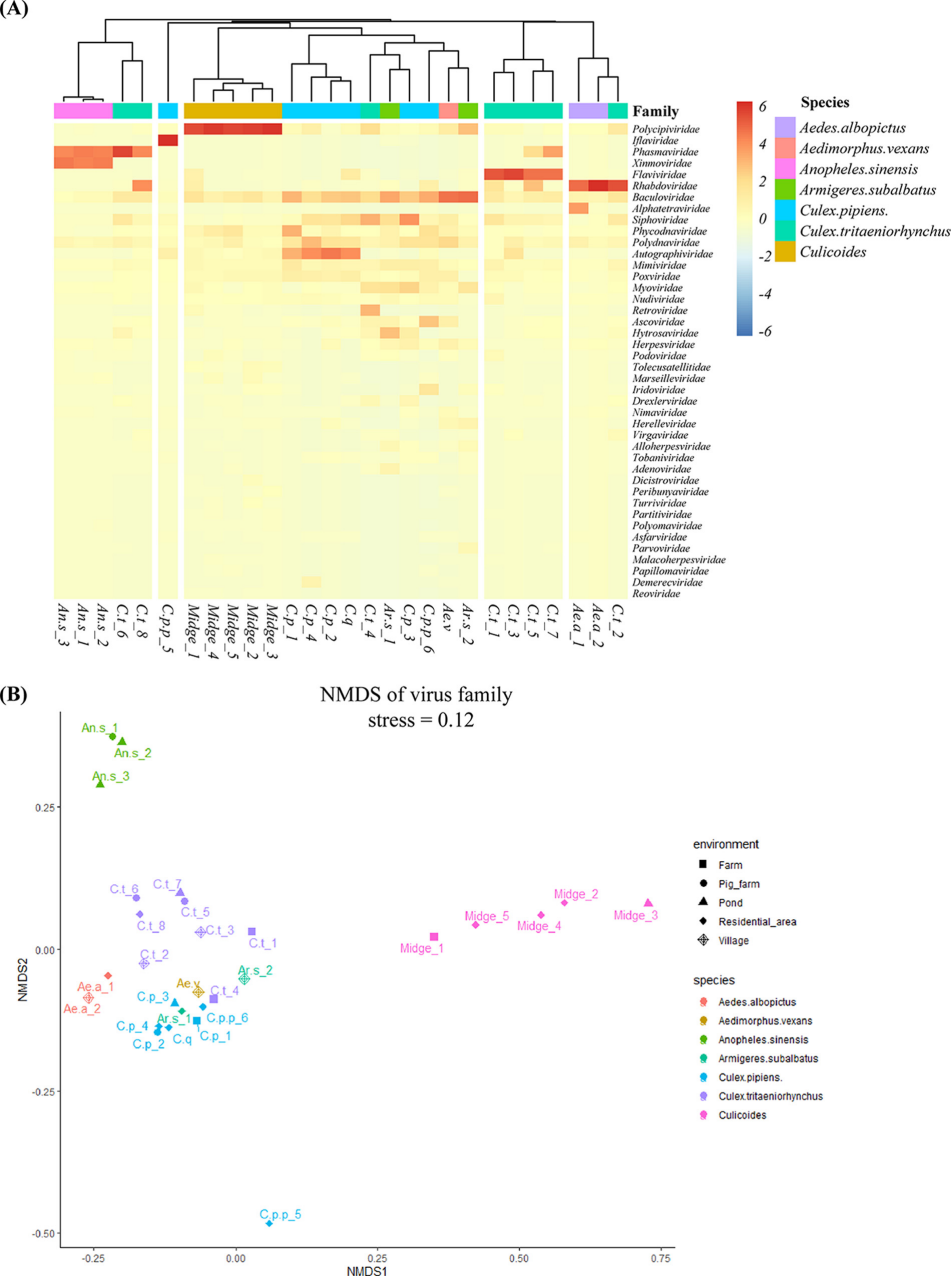
The relationship between virome, host species, and habitat environment. (A) Heatmap of sample clustering based on RPM. The heatmap shows the normalized reads counts by meta-viromic Seq on the log_2_ scale. The hierarchical clustering is based on the Euclidean distance matrix calculated from the normalized reads count. The viral family shown in the heatmap are from the taxonomic annotation by Kraken2 and Bracken. (B) Nonmetric multidimensional scaling (NMDS) of virus on family level. Using Bray-Curtis dissimilarities calculated from the abundance of virome, NMDS was then applied to the ordination analysis for the beta diversity. STRESS = 0.1234, PERMANOVA test on species: *P* = 0.001 and R^2^ = 0.6129.

Our data suggested that the virome composition is species related and that different species have different core virome groups, especially *Culicoides* (Polycipiviridae), *Ae. albopictus* (Rhabdoviridae), and *A. sinensis* (Phasmaviridae, Xinmoviridae; Fig. 2B and 3A). The virome composition had little to do with the habitat environment (stress = 0.12; Fig. 3B). Baculoviridae, Mimiviridae, Poxviridae, Polydnaviridae, and Siphoviridae were identified in all *C. pipiens* samples. One *C. p. pallens* sample (C.p.p_5) contained a large number of Iflaviridae reads and was obviously an outlier, probably because the mosquitoes were full of blood (Fig. 2B and 3). Thirty-eight viral families were found in *C. tritaeniorhynchus*, and 19 of them were common to all habitats.

### Virome characterization for important arboviruses and ISVs from *C. tritaeniorhynchus*, *Ae. Albopictus*, and *Culicoides*.

Among all 10 viral taxonomy, single-strand (ss)RNA^+^ were annotated with the longest viral contigs, followed by ssRNA^–^, double-strand (ds)DNA, and dsRNA. The major viral genome types varied among the sampled speices. For example, the major type in *Culicoides* and *Ae. albopictus* was ssRNA^+^, whereas that in *A. sinensis* was ssRNA^–^. Among all samples, we annotated 14 viruses with vertebrate hosts (Table S3).

**FIG 4:**
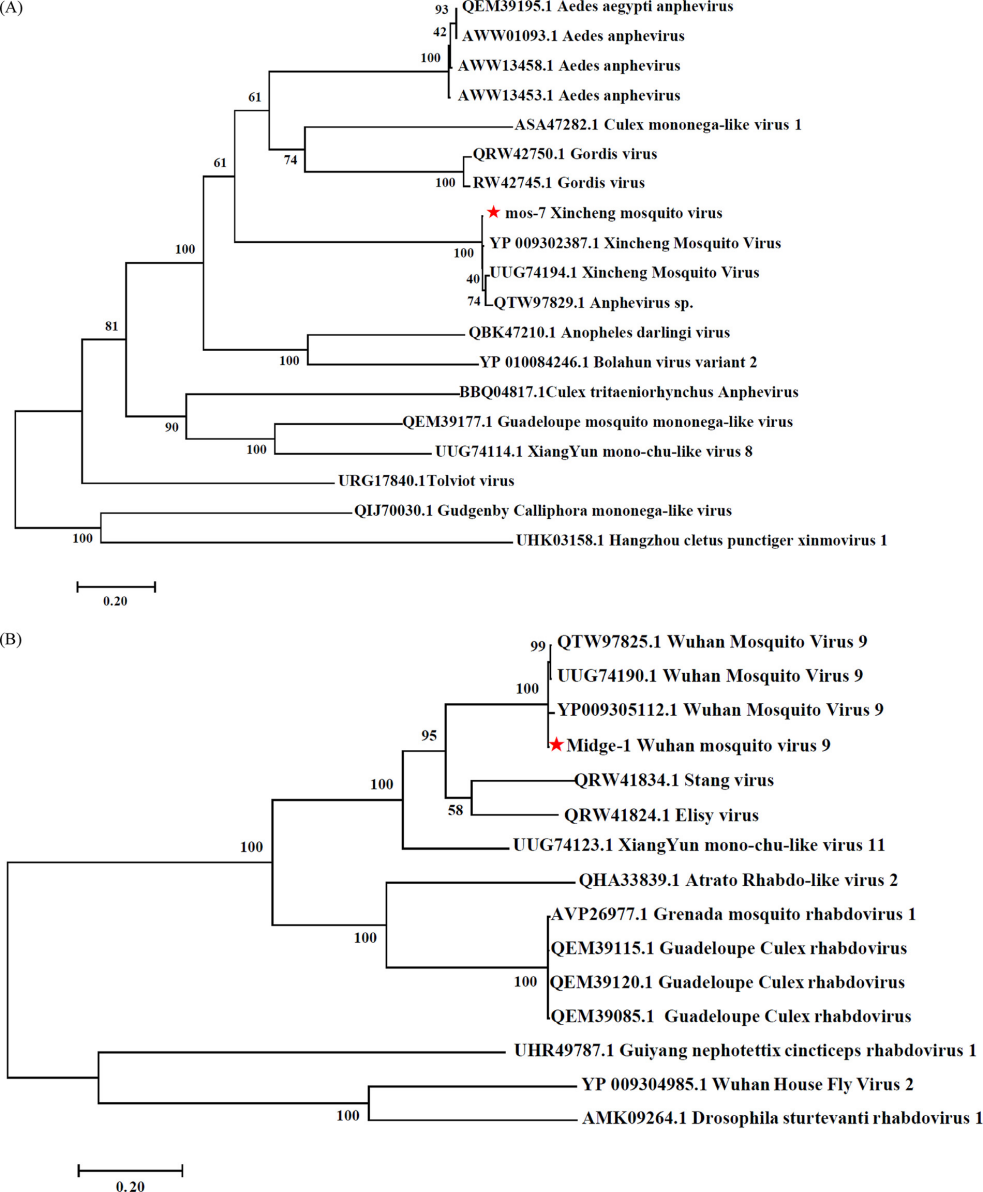
Phylogenetic tree of virus in mosquitoes and *Culicoides* samples. (A) Maximum likelihood phylogeny of Wuhan mosquito virus 9 based on the amino acid sequence of RdRp gene. (B) Maximum likelihood phylogeny of Xincheng mosquito virus based on the amino acid sequence of RdRp gene.

Previous studies have shown that arboviruses are concentrated in six viral families: Flaridae, Rhabdoviridae, Peribunyaviridae, Togavividae, Reoviridae, and Phenuivividae (40). We analyzed contig data from these six families. West Nile virus, dengue virus 3, High Island virus (Reoviridae), Guapiacu virus (Flaviviridae), and Piry virus (Rhabdoviridae) sequences were found in *Ae. albopictus*. Some contigs were found to have 80% to 100% aa identity with the NS5 region of dengue virus 3 (GenBank: MH051731.1; Fig. S1) and 80% identity with that of Aedes aegypti, suspected to reflect endogenous viral elements or part of the *Aedes* genome. No viral gene was found in the sequences annotated as dengue virus and West Nile virus, and the corresponding PCR results were negative. In *Culicoides*, Quang Binh virus (Flaviviridae), Wuhan mosquito virus 9 and *Culex tritaeniorhynchus* rhabdovirus (Rhabdoviridae), and a novel Thimiri orthobunyavirus (Bunyaviridae) were identified. A small amount of *Culex* flavivirus was found in *Culicoides* and *C. tritaeniorhynchus*. Wuhan mosquito virus 9 was found in midges and mosquitoes, and its sequence included open reading frames (ORFs) 1 to 3, glycoprotein, and RNA-dependent RNA polymerase (RdRp). The conserved domains include Mononeg_RNApol and paramyx_RNAcap. Phylogenetic analysis based on the RdRp showed the closest affinity to Wuhan mosquito virus 9 (GenBank: YP009305112.1; Fig. 4B). The whole Xincheng mosquito virus genome, including Mononeg_mRNAcap and paramyx_RNAcap, was obtained from *A. sinensis* by de-novo assembly (Fig. 4A). A long (9,751 bp) contig with 81.69% nucleotide identity with Culex densovirus was found in *Armigeres subalbatus*. Only one viral protein, Aedes aegypti Thai densovirus nonstructural protein 1, was found, and no conserved domain was detected. Although the RdRp fragment could not be found and whether it was a new virus could not be confirmed, the ability of some densovirus to reduce mosquito populations deserves attention (41). We also found a 3,071-bp contig annotated as Aedes pseudoscutellaris reovirus with 70.43% nucleotide identity and 59.02% aa identity in the VP5 region in *C. pipiens*.

### Source of blood meal from mosquitoes.

We analyzed blood meals in five pooled mosquito samples from four sampling sites. We compiled their corresponding Chordata contigs and chose animals that mosquitoes and *Culicoides* might feed on. The blood meal sources fell into four broad categories of Aves and Mammalia (chicken, duck, pig, and human) that were broadly consistent with the habitats where the mosquitoes were collected. Blood meals from *A. subalbatus* samples from the village and *Ae. albopictus* samples from the residential area were mainly human, and that of *C. p. pallens* from the residential area was mainly duck. Those of *C. tritaeniorhynchus* collected from the farmland and pig farm were chicken and pig, respectively. Rodent sequences were found in various samples, potentially representing a link in the mosquito-borne virus transmission chain. The viruses carried by the blood-sucking mosquitoes can be carried by rats, chickens, pigs, and humans, concordant with the blood meal calls.

### Potential discovery of novel viruses and dark matter.

**FIG 5:**
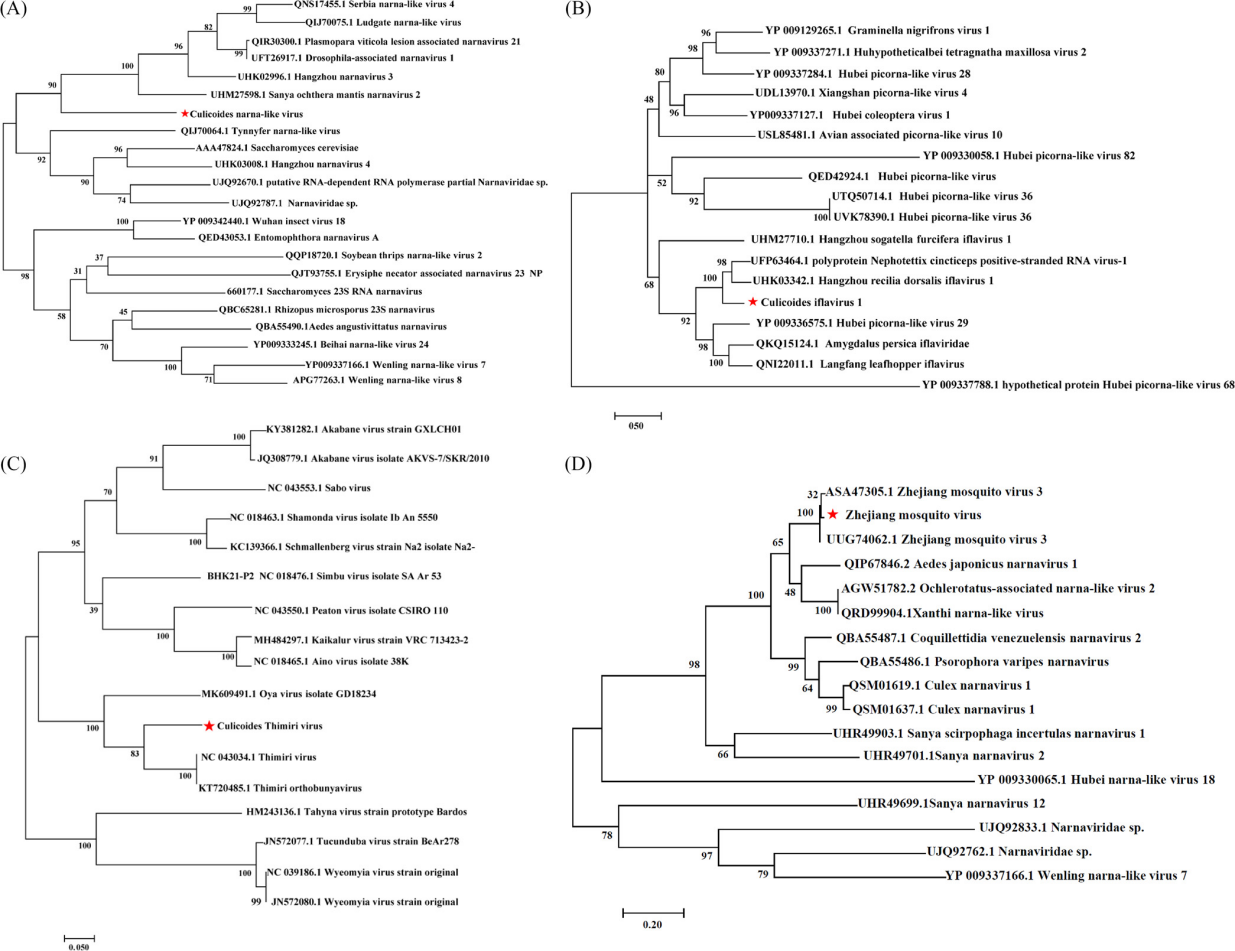
Phylogenetic tree of potential novel virus in mosquitoes and *Culicoides* samples. (A) Maximum likelihood phylogeny of unclassified Narnavirus based on the amino acid sequence of part RdRp gene. (B) Maximum likelihood phylogeny of unclassified iflavirus based on the amino acid sequence of polyprotein. (C) Maximum likelihood phylogeny of unclassified Orthobunyavirus based on the amino acid sequence of part segment L. (D) Maximum likelihood phylogeny of Zhejiang mosquito virus 3 based on the amino acid sequence of RdRp gene.

De-novo read assembly revealed the presence of a novel narnavirus (Narnaviridae) in two *Culicoides* samples (Midge_3 and Midge_4), which was named *Culicoides* narna-like virus 1 (CNV1). The longest ORF in the 2,485-bp genome of CNV1 encodes an RdRp related closely to Sanya ochthera mantis narnavirus 2 (36.36% aa similarity; Table S4a). The narnavirus hosts phylogenetically closest to CNV1 are arthropods, including *Ochthera mantis* and *Drosophila* (Fig. 5A). Many Simbu serogroup virus (*Orthobunyavirus*, Bunyaviridae) sequences were found in the Midge_1 and Midge_3 samples. Parts of the L segment (4,186 bp, ~81.27% nucleotide identity with MH484336.1) and S segment (982 bp, ~85.35% nucleotide identity with MH484338.1) were obtained by de-novo assembly, and the L segment contained a conserved domain annotated as the Bunya RdRp. Phylogenetic analysis showed that it was closest to *Thimiri orthobunyavirus* (Fig. 5C). We also assembled a contig (10,785 bp) annotated as Hubei picorna-like virus 29 (73.10% nucleotide identity), with 44.12% identity of the hypothetical protein. Three conserved domains were identified using the NCBI CD-Search software with an E-value of e^−5^: ps-ssRNAv-Picornavirales (catalytic core domain of RdRp), rhv_like (Picornavirus capsid protein domain_like), and RNA_helicase (Table S5). A phylogenetic tree constructed based on a polyprotein (3,156 aa) suggested that the contig represented a novel *Iflavirus*, a picorna-like virus that infects arthropods (Fig. 5B). Another contig was annotated as Hubei tombus-like virus 20 and was considered to be a new viral sequence because it showed <80% nucleotide identity. A 3,323-bp novel viral sequence of Zhejiang mosquito virus 3 was found in *C. pipiens* and showed 93.01% nucleotide identity with the reference genome (KX883461.1 3226 bp). Phylogenetic analysis showed that the RdRp gene was related most closely to Zhejiang mosquito virus 3 (ASA47305.1; Fig. 5D), followed by some narnaviruses, such as Aedes japonicus narnavirus 1 (QIP67846.2) and Xanthi narna-like virus (QRD99904.1).

**FIG 6:**
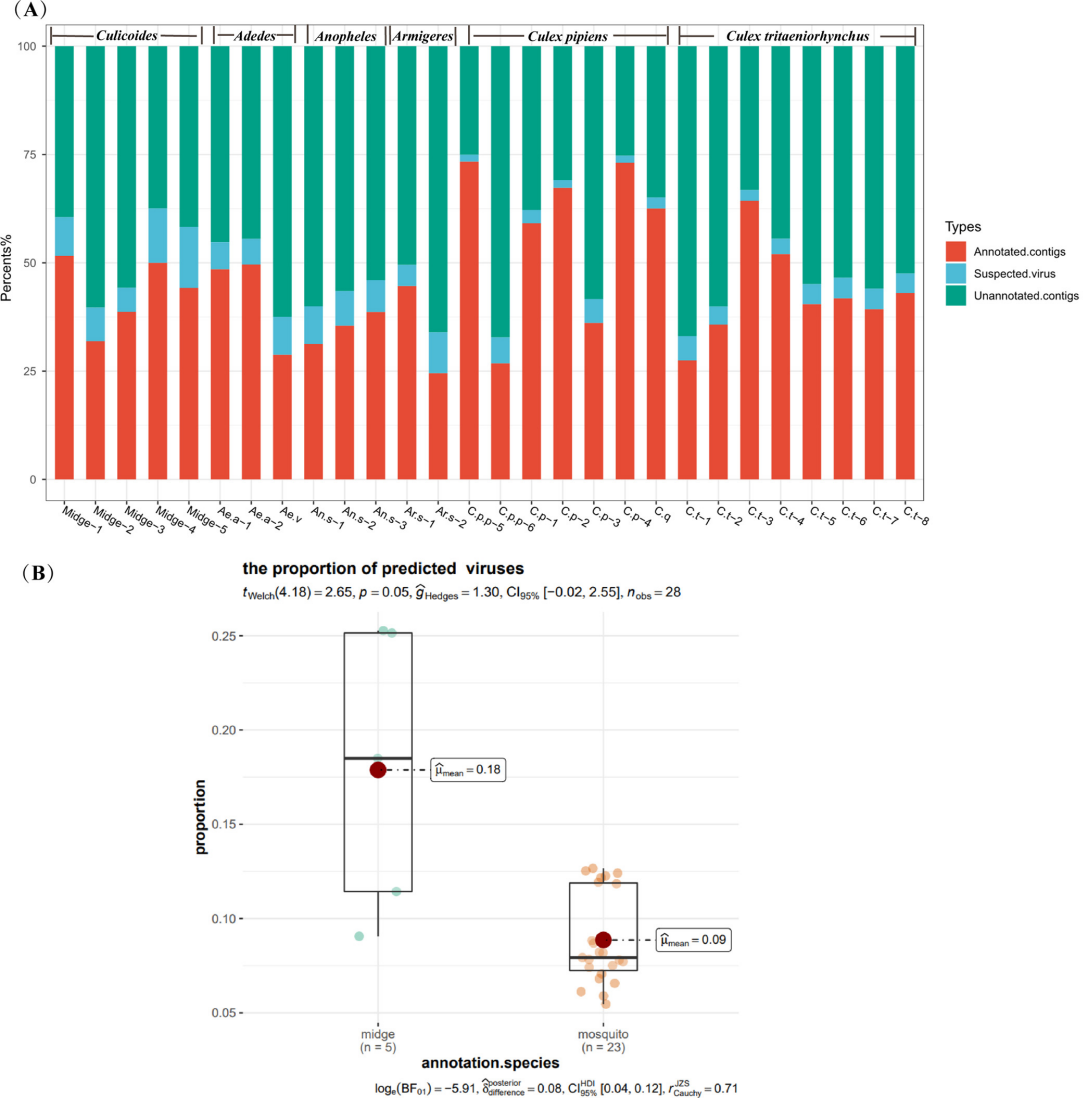
Comparison of the proportion of viral unblast contigs in mosquitoes and *Culicoides*. (A) The proportion of the annotated contigs. (B) The comparison of annotated contigs between mosquitoes and *Culicoides*. Welch’s *t* test was used for comparison between mosquitoes and *Culicoides*.

Nearly 50% (*n *= 2,704,391) of the contigs were unannotated; 2,180,762 were annotated (Fig. 6A). The proportions of suspected viral sequences predicted by DeepVirFinder (DVF) varied widely among samples and ranged from 5.46% (for C.p_2) to 25.27% (for Midge_5). The proportion of unannotated contigs predicted to be viral sequences was larger in *Culicoides* than in mosquito samples (Fig. 6B). The median length of the sequences identified by BLAST was 421 to 794 bp and that of the sequences predicted by DVF was 578 to 887 bp (Table S6). The GC content of sequences identified by BLAST ranged from 35% to 49% (mean, 42.7%) and that of sequences predicted by DVF ranged from 36% to 47% (mean, 43.5%), with no significant difference between methods (*P* = 0.18, *n *= 28; Fig. S2).

## DISCUSSION

This study is the first in which high-throughput sequencing was used to characterize the viruses carried by mosquitoes and midges on Zhoushan Island, contributing to the knowledge of arthropod viromics in the coastal areas of southeastern China. Similar to previous findings, we found that *C. tritaeniorhynchus* and *Ae. albopictus* were the dominant mosquito species and had preferred habitats (42), and *C. arakawae* is a common local *Culicoides* species (43).

Viruses of the Rhabdoviridae, Polycipiviridae, Phasmaviridae, Phenuiviridae, and other virus families were identified in this study, as in other studies, but in markedly different proportions (32). In this study, Polycipiviridae viruses (e.g., Hubei picorna-like virus 82, a ssRNA^+^ insect-related virus found in spiders) were very abundant in *Culicoides* viromes, which has not been reported previously. Meta-viromic sequencing of *C. impunctatus* viromes revealed viruses of the Polycipiviridae family, but in only one group (33). We also made the novel identification of *Thimiri orthobunyavirus* (genus *Orthobunyavirus*), originally isolated in birds (44) and known to be hosted by *Culicoides histrio* (45), in *Culicoides* collected from the farm and pond sites, suggesting that this virus exists in different environments and organisms in the study region. To our knowledge, this is the first description of Simbu serogroup viruses in *Culicoides* from Zhoushan Island. Orthobunyaviruses of the Simbu serogroup are transmitted by insects (primarily biting midges) and infect mammals and birds (46). Recent research suggests that *Culicoides* are associated with potential Simbu serogroup virus transmission and continued prevalence in livestock (46–49). Thus, further epidemiological investigation of Simbu serogroup viral infection in the study region is needed. Our research has expanded the group of known *Culicoides*-associated viruses and demonstrated that much about these viruses remains unknown.

Different mosquito species may carry unique virus groups (24, 29), which will help to strengthen the monitoring and research on important mosquito species that carry insect-borne viruses. The core viromes identified in this study were very diverse among mosquito species and different than those reported previously (28, 50); the eukaryotic viromes of mosquitoes and *Culicoides* collected on Zhoushan Island are stable and diverse (27–29). More Hubei mosquito virus 2 was found in *C. tritaeniorhynchus*. Wuhan mosquito virus 1 (Phasmaviridae) and Xincheng mosquito virus (Xinmoviridae) were found in *A. sinensis* and a relatively complete Xincheng mosquito virus was assembled. Similar results were obtained in previous mosquito virome studies conducted in Hubei Province, China (25, 51), but not in studies of the same mosquito species conducted on other continents (28). Species-specific mosquito viromes may be stable within regional ecosystems, as core virome genomes are usually found in relatively small areas (e.g., Guadeloupe, Zhoushan) or large geographic areas (e.g., Australia, Northern Europe, and parts of China) are conservative and stable (28). The Xincheng mosquito virus that we found is not completely consistent with that found in *A. sinensis* in Hubei Province (~96.7% nucleotide similarity), and other viruses have similar findings, reflecting small differences in the core viromes of mosquito species at this geographic scale.

In this study, we extended the scope of molecular approaches to identify blood meal sources using meta-viromic sequencing data (52). The host identification was broadly consistent with the habitats where the samples were collected, which enables xenosurveillance. A long contig of Avian leukosis virus was found in a group of apparently blood-sucking *C. tritaeniorhynchus* mosquitoes, for which the blood meal source was identified as chickens on a farm. This fragment accounts for 70% of the full length of the virus (GenBank no. MK951780.1). As viral proteins were found in it, we consider it to be an exogenous viral sequence. When the virus is incorporated into the mosquito's blood meal during feeding, the meta-viromic of satiated mosquitoes can reflect the viral diversity of many mammals, birds, and humans by means of xenosurveillance (53–55). We believe that xenosurveillance for viruses with vertebrate hosts in the viromes of mosquitoes and midges could play an important role in the monitoring of local vertebrate diseases.

Genome sequencing has enhanced our understanding of emerging viromes by providing blueprints of the evolutionary and functional diversity of viruses, but sequences always contain dark matter that cannot be identified or matched. As not all viruses can be isolated and cultured in the laboratory, numerous algorithms, databases, and pipelines have been developed to process virome sequencing data and support dark matter exploration (56–58). Using DVF and CheckV software, we performed a preliminary exploration of a large number of unannotated sequences in the study samples, looking for possibly viral sequences in the dark matter. The dark matter contained a certain proportion of viral sequences, with a larger proportion of unannotated sequences predicted to be viruses in midge than in mosquito samples. A fraction of the ORFs was annotated as viral proteins with <50% similarity. We speculate that midges carry a large number of unknown viruses. Further exploration is needed to confirm this speculation and examine virus evolution in these species.

This study has some limitations. Due to the COVID-19 pandemic, we were able to collect samples at only a small number of sites, and not continuously in all cases. Moreover, although meta-viromic sequencing aids the detection of less abundant viruses, it does not enable the distinction of endogenous viral elements; thus, further analysis and validation are needed to enhance the robustness of the data. We will continue to monitor the viromes of mosquitoes and biting midges on Zhoushan Island and other islands off China’s southeastern coast.

## MATERIALS AND METHODS

### Sample collection and species identification.

Zhoushan Island is located in northeast Zhejiang Province, China. It has a subtropical monsoon climate with multiple typhoons occurring in summer, which facilitates the efficient reproduction of mosquitoes, *Culicoides*, and the viruses they carry. Imported cases of dengue fever on Zhoushan Island have been reported, and outbreaks of dengue fever have occurred in nearby Daishan County (59). From June 2021 to September 2021, we collected samples of mosquitoes and *Culicoides* at five locations on Zhoushan Island: a pig farm, farmland, a pond, a village, and an urban residential area (Fig. 1A; Table S1). The samples were collected using UV traps. Captured mosquitoes and *Culicoides* were stored immediately at −20°C, and then identified by experienced field biologists based on their morphological characteristics under an anatomical lens while on ice. The samples were then transported to the laboratory in a freezer, and the mosquito species were confirmed by sequencing of the cytochrome c oxidase subunit I gene (60). All samples were then stored at −80°C until further analysis.

### RNA extraction.

One to 10 female mosquitoes or 20 to 50 *Culicoides* were pooled based on species, sampling location, and visible blood engorgement of the mosquitoes. When more than 10 mosquitoes or 50 midges had been collected from a sampling site, 10 or 50 were selected randomly. Each pooled sample was washed once with sterile phosphate-buffered saline solution and then twice in diethyl pyrocarbonate (DEPC)-treated water to remove potential surface microorganisms. RNA was dissolved in 30 μL non-RNase DEPC and frozen at −80°C.

### cDNA library preparation, amplification, and sequencing.

DNA library construction and sequencing were performed at the Beijing Genomics Institute. A nucleic acid microbes purification kit (BGI PathoGenesis Pharmaceutical Technology, BGI-Shenzhen, Shenzhen, China) was used to enrich the viruses in all samples. The remaining RNA was fragmented to about 280 bp and then reverse transcribed into cDNA, followed by second-strand synthesis. Using the synthetic double-stranded DNA, a DNA library was constructed through end repair, unique dual-index adaptor ligation, and PCR amplification. The library was qualified with a Qubit 4.0 fluorometer (Thermo Fisher Scientific, Foster City, CA, USA) and the fragment size was analyzed with a Qsep100 system (Hangzhou Houze Biotechnology Co., Ltd.). After circularization and DNA nanoball generation, the resulting libraries were sequenced with an MGI high-throughput sequencing set (PE150) on MGISEQ-2000/T7 platforms (MGI, Shenzhen, China; Fig. 1B).

### Bioinformatics analysis.

After the removal of adapter, low-quality, and low-complexity reads, high-quality genome sequencing data were generated with the fastp software (v. 0.20.0) (61). Clean reads were delivered for the identification of microorganisms through meta-viromic next-generation sequencing analysis. The reads were first mapped against the host sequence database to remove host genome sequences using hisat2 (v. 2.1.0) (62) (Table S2). The MEGAHIT software (v. 1.1.2) (63) was used to assemble reads into a scaffold sequence. A nonredundant set was compiled by clustering nucleic acids with ≥90% identity using CD-HIT (v. 4.8.1, cd-hit-est mode) (64).

### Viral sequence identification.

Host-removal filtering reads were used to identify microorganisms with Kraken2 (v. 2.1.2) (62) and the Refseq and NR databases. We used Bracken (v. 2.6) (65) to acquire a highly accurate statistical abundance of species. The BWA software (v. 0.7.17) (63) was used to map the reads into a genome. To understand the distribution of species within the microbial cargo of the mosquitoes and *Culicoides*, we first examined the overall proportions of nonhost reads that could be assigned to viral, bacterial, eukaryotic, and archaeal organisms. BLAST (v. 2.5.0+, default parameters and E-value threshold of 10^−5^) was used to align the scaffold sequence with the NT database. Then, the sequences annotated as viruses were extracted and evaluated using CheckV software (v. 1.0.1) (66). To identify endogenous viral sequences, we used NCBI BlASTp searches for eukaryotes, bacteria, and archaea (NCBI taxonomy IDs 2759, 2, 2157) with the default parameters and E-value threshold of 10^−5^. We also performed online BLASTn alignment for retroviral sequences. Relationships between identified viruses and their hosts were determined based on NCBI taxonomy ID pairs in the Virus-Host database published on March 15, 2022 (https://www.genome.jp/virushostdb/) (67). Viral proteins were predicted using the “meta” pattern in Prodigal (v. 2.6.3) (68).

### Viral structure, conserved domain, and phylogenetic analyses.

All proteins were annotated by the NCBI nr database using BLASTp (E-value threshold of 1E-10). The threshold of identity for new sequences was set with amino acid (aa) sequences < 90% or nucleotide sequences < 85%. Conserved domains were searched for using the NCBI CD-Search tool (http://www.ncbi.nlm.nih.gov/Structure/cdd/wrpsb.cgi) (69). Maximum-likelihood phylogenetic trees were produced using MEGA 7 (70) with 1,000 bootstrap replicates.

### Exploration of viral sequences in dark matter.

To annotate potential viral sequences among unclassified sequences, DeepVirFinder (DVF, v. 1.0) (56) was run with the Python script, and contigs (>500 bp) with scores > 0.9 and *P* < 0.05 were considered to be viral (Fig. 1C). Sequences annotated as viruses were extracted and evaluated using CheckV (66). Proteins were predicted using the “meta” pattern in Prodigal (v. 2.6.3).

### PCR verification.

To verify the presence of dengue virus and West Nile virus (71) in the samples, conventional PCR was performed using the FastKing One-Step RT-PCR kit (TIANGEN Biotech, China). The Baltimore virus classification system was used with BLAST based on the ICGT database of the International Committee on Taxonomy of Viruses (ICTV, https://talk.ictvonline.org/).

### Data availability.

The raw sequencing data sets for the current study are available in the NCBI Sequence Read Archive repository, under the Bioproject with accession code PRJNA847438 (NCBI BioProject database, www.ncbi.nlm.nih.gov/bioproject/847438).
