# Supplementary material for: Meta-Viromic Sequencing Reveals Virome Characteristics of Mosquitoes and Culicoides on Zhoushan Island, China
Source: Microbiol Spectr. 2023 Jan 18;11(1):e02688-22. doi: 10.1128/spectrum.02688-22 (PMC9927462; doi:10.1128/spectrum.02688-22)
Supplement: Supplemental file 8 — Fig. S1 and S2. Download spectrum.02688-22-s0008.pdf, PDF file, 0.1 MB [file spectrum.02688-22-s0008.pdf]

Supplementary Material for

Meta-viromic sequencing reveals virome characteristics of mosquitoes and Culicoides in Zhoushan Island, China.

Table S1. Basic information of several sampling sites.

Table S2. The host reference genome.

Table S3. The combined result of BLASTn and CheckV.

Table S4a. The BLASTp result of viral sequences' ORF (viral database).

Table S4b. The BLASTp result of viral sequences' ORF (Eukaryotic and Bacteria database).

Table S5. The conserved domains annotated by CD-search.

Table S6. The characteristic of viral sequences annotated by DVF and BLASTn.

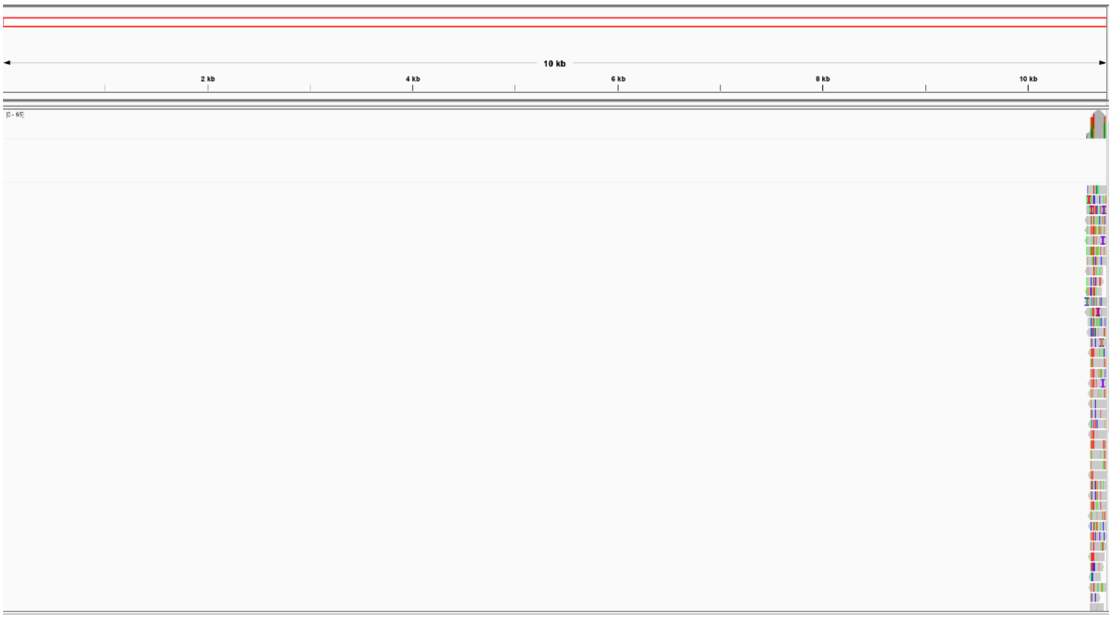

Figure S1. The assembly results of dengue virus 3 in the sample.

Sample reads were mapped to the dengue virus 3 genome (GenBank: MH051731.1) using BWA software and visualized using IGV software.

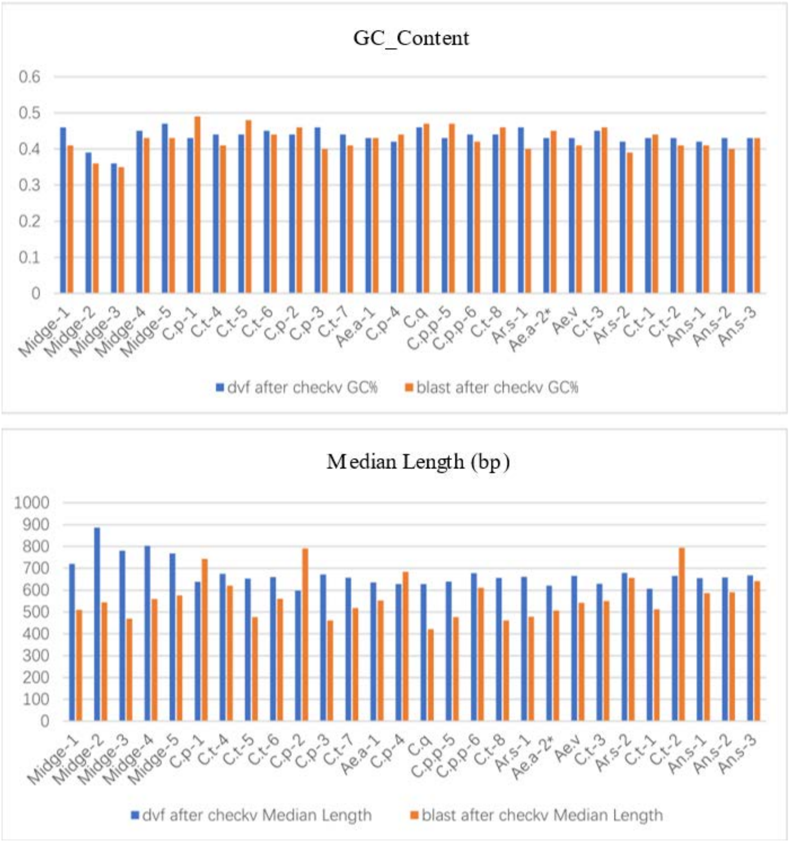

Figure S2. Characteristics of viral sequences identified by BLAST and DeepVirFinder.
